# The devil you know and the devil you don’t: current status and challenges of bovine tuberculosis eradication in the United States

**DOI:** 10.1186/s13620-023-00247-8

**Published:** 2023-07-25

**Authors:** Daniel J. O’Brien, Tyler C. Thacker, Liliana C. M. Salvador, Anthony G. Duffiney, Suelee Robbe-Austerman, Mark S. Camacho, Jason E. Lombard, Mitchell V. Palmer

**Affiliations:** 1 Michigan Department of Natural Resources, Wildlife Disease Laboratory, 4125 Beaumont Road, Room 250, Lansing, MI 48910-8106 USA; 2 Retired. Current address: Department of Fisheries and Wildlife, Michigan State University, 480 Wilson Road, East Lansing, MI 48824 USA; 3 United States Department of Agriculture, Animal and Plant Health Inspection Service, National Veterinary Services Laboratories, 1920 Dayton Avenue, Ames, IA 50010 USA; 4 Institute of Bioinformatics, Center for the Ecology of Infectious Diseases, Department of Infectious Diseases, College of Veterinary Medicine, University of Georgia, Athens, GA 30602 USA; 5 School of Animal & Comparative Biomedical Sciences, University of Arizona, Shantz Building, 1177 E 4th St, Tucson, AZ 85719 USA; 6 United States Department of Agriculture, Animal and Plant Health Inspection Service—Wildlife Services, 2803 Jolly Road, Suite 100, Okemos, MI 48864 USA; 7 United States Department of Agriculture, Cattle Health Center, Animal and Plant Health Inspection Service—Veterinary Services, Centennial Campus, Raleigh, NC 27606 USA; 8 United States Department of Agriculture, Field Epidemiologic Investigation, Animal and Plant Health Inspection Service, Veterinary Services, 2150 Centre Avenue, Bldg. B, Fort Collins, CO 80526 USA; 9 United States Department of Agriculture, Agricultural Research Service, National Animal Disease Center, 1920 Dayton Avenue, Ames, IA 50010 USA

**Keywords:** Bovine tuberculosis, Eradication, Farm biosecurity, Livestock, Management, *Mycobacterium bovis*, *Odocoileus virginianus*, White-tailed deer, Whole genome sequencing, Wildlife

## Abstract

Having entered into its second century, the eradication program for bovine tuberculosis (bTB, caused by *Mycobacterium bovis*) in the United States of America occupies a position both enviable and daunting. Excepting four counties in Michigan comprising only 6109 km^2^ (0.06% of US land area) classified as Modified Accredited, as of April 2022 the entire country was considered Accredited Free of bTB by the US Department of Agriculture for cattle and bison. On the surface, the now well-described circumstances of endemic bTB in Michigan, where white-tailed deer (*Odocoileus virginianus*) serve as a free-ranging wildlife maintenance host, may appear to be the principal remaining barrier to national eradication. However, the situation there is unique in the U.S., and far-removed from the broader issues of bTB control in the remainder of the country. In Michigan, extensive surveillance for bTB in deer over the last quarter century, and regulatory measures to maximize the harvest of publicly-owned wildlife, have been implemented and sustained. Prevalence of bTB in deer has remained at a low level, although not sufficiently low to eliminate cattle herd infections. Public attitudes towards bTB, cattle and deer, and their relative importance, have been more influential in the management of the disease than any limitations of biological science. However, profound changes in the demographics and social attitudes of Michigan’s human population are underway, changes which are likely to force a critical reevaluation of the bTB control strategies thus far considered integral. In the rest of the U.S. where bTB is not self-sustaining in wildlife, changes in the scale of cattle production, coupled with both technical and non-technical issues have created their own substantial challenges. It is against this diverse backdrop that the evolution of whole genome sequencing of *M. bovis* has revolutionized understanding of the history and ecology of bTB in Michigan, resolved previously undiscernible epidemiological puzzles, provided insights into zoonotic transmission, and unified eradication efforts across species and agencies. We describe the current status of bTB eradication in the U.S., how circumstances and management have changed, what has been learned, and what remains more elusive than ever.

## Background

Having now entered into its second century [1, 2], the eradication program for bovine tuberculosis (bTB, caused by *Mycobacterium bovis*) in the United States of America occupies a position at once both enviable and daunting. With the exception of four counties in Michigan comprising only 6109 km^2^ (0.06% of US land area) which were classified as Modified Accredited (MA), as of April 2022 the entire country was considered Accredited Free (AF) of bTB by the US Department of Agriculture (USDA) for cattle and bison (*Bison bison*). All 50 states were classified as MA, two steps below AF, for captive cervids. Between 1 October 2018 and 30 September 2019, only 10 cattle herds were detected as infected with *M. bovis*; 4 of those were in Michigan. Considering where the US bTB eradication program began in 1917, with 5% of US cattle tuberculous on average [2], an estimated 25,000 to 50,000 human deaths per year attributed to bTB [1], and an estimated US$13-$55 billion annual economic return through 2003 [3], the progress and benefit of the eradication program, coupled with milk pasteurization, is astonishing. On the surface, the now well-described circumstances of endemic bTB in Michigan, where white-tailed deer (WTD; *Odocoileus virginianus*) serve as a free-ranging wildlife maintenance host [4–8], may appear to be the principal remaining barrier to national eradication. However, the situation there is unique in the US, and in some respects relatively far-removed from the broader issues of bTB control in the remainder of the country.

In Michigan, extensive surveillance for bTB in free-ranging white-tailed deer over the last quarter century by the Michigan Department of Natural Resources (MDNR) has established a consistent, reliable metric with which to monitor the reservoir of infection in the host population over time. Regulatory measures designed to maximize the harvest of publicly-owned wildlife to minimize deer densities and other putative spillover hosts of *M. bovis* have been implemented and sustained. Prevalence of bTB in deer has remained at a low level as a result, although not sufficiently low to eliminate cattle herd breakdowns in the endemic area [9]. Public attitudes about bTB, cattle and deer, and their relative importance have arguably been more influential in the management of the disease than any limitations of biological science [10–12]. However, profound changes in the demographic characteristics and social attitudes of Michigan’s human population are underway, changes which are likely to force a critical reevaluation of the bTB control strategies thus far considered integral.

More broadly, in the rest of the U.S. where bTB is not self-sustaining in wildlife, changes in the scale of cattle production, coupled with both technical (e.g., limitations in diagnostic tests, deficiencies in trace methodologies) and non-technical (e.g., antiquated regulations, shrinking fiscal resources for bTB eradication programs) issues have created their own substantial challenges. Both livestock health regulators and the animal industries themselves have recognized the need for changes in the structure of the federal bTB eradication program. To that end, federal animal health officials have entered negotiations with the states and industry leaders to develop standards-based regulations, transitioned from predominantly herd depopulation to test-and-remove as a routine response to dairy herd breakdowns, and substantially restricted importation of cattle of unverifiable bTB status. Yet disagreements concerning the details of standards-based regulations for the states have delayed promulgation.

It is against this diverse backdrop that the evolution of whole genome sequencing (WGS) of *M. bovis* has become a tool that has revolutionized understanding of the history and ecology of bTB in Michigan, resolved previously undiscernible epidemiological puzzles associated with cattle herd breakdowns on a continental basis, provided insights into zoonotic transmission and evolution of the pathogen, and unified eradication efforts across species and agencies. Yet many of the fundamental challenges of eradication remain substantially untouched by such technological advances. Here, we describe the current status of bTB eradication in the US, how circumstances and management have changed in the recent past, what has been learned, and what remains more elusive than ever.

### The devil you know: Perpetual management of bTB in Michigan

#### Wildlife: surveillance and monitoring

**Fig. 1:**
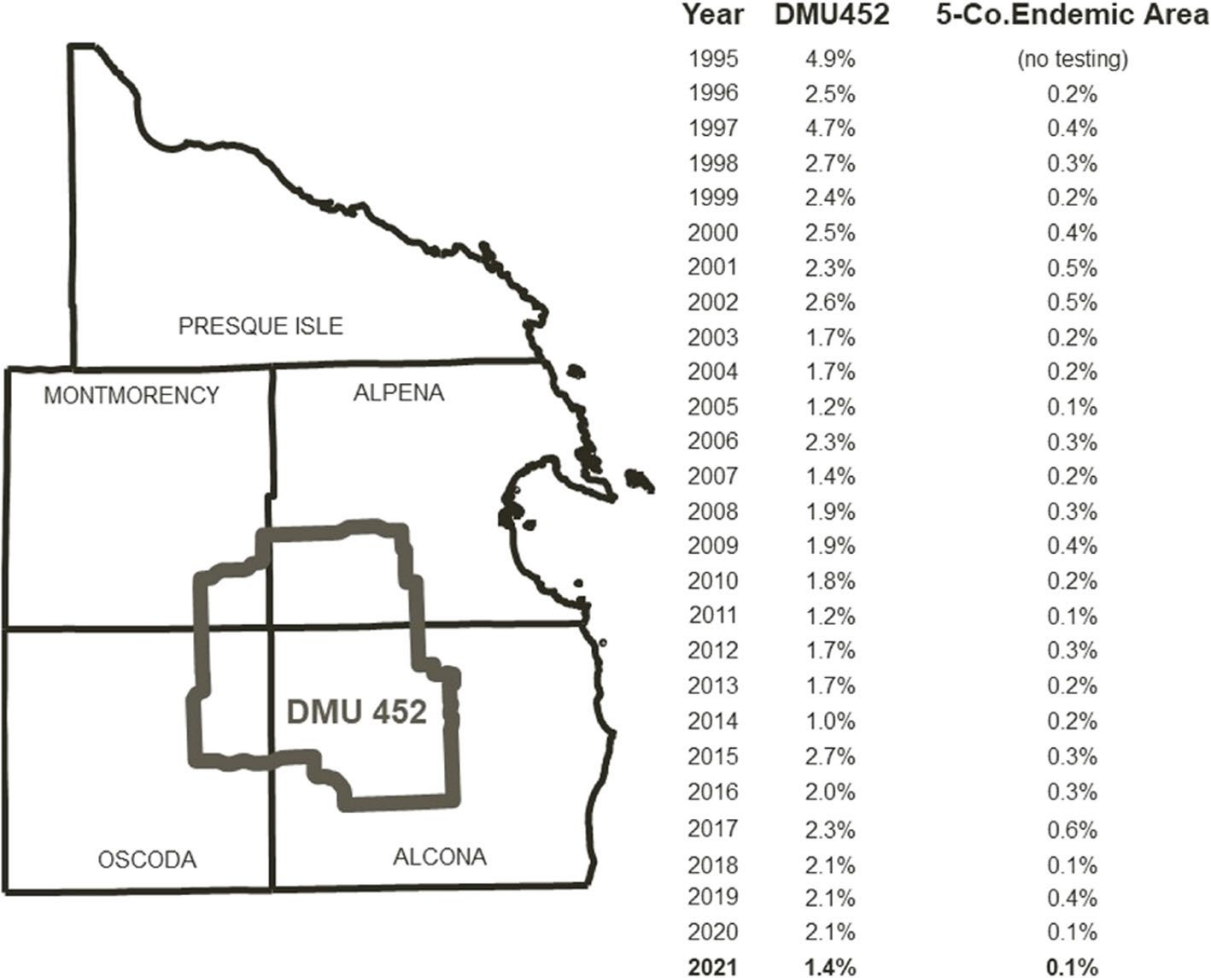
Apparent prevalence of bovine tuberculosis in free-ranging white-tailed deer, Deer Management Unit 452, and the remainder of the five county endemic area outside it, Michigan, USA, 1994–2021

The year 2014, which saw the last International *M. bovis* Conference in Cardiff, UK, also saw the reservoir of bTB in WTD reach its lowest point since systematic surveillance began a quarter century ago, 1% as measured by apparent prevalence in the core outbreak area, Deer Management Unit (DMU) 452 in Michigan’s northeastern Lower Peninsula ([13], Fig. 1). While a marked reduction from the 4.9% recorded at the outset of systematic surveillance in 1994, since 2014, annual period prevalence has since remained above that level, as noted in Fig. 1. In the remainder of the five county area outside the core where bTB is endemic in deer, prevalence has remained relatively stable over the same period at approximately an order of magnitude lower than prevalence in the core.

**Fig. 2:**
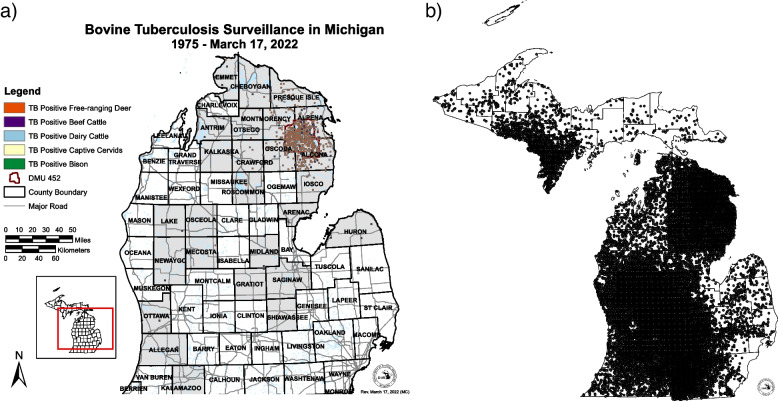
**a** Scale map of Michigan, USA’s, Lower Peninsula showing Sect. (1 mi^2^, ~ 2.6 km^2^) locations of all bTB-positive animals of all species found since 1975. Counties shaded grey have had at least one infected animal. **b** Map showing testing effort for bTB in free-ranging white-tailed deer, 2015 to 2021. Dots indicate site of at least one animal tested

Perhaps remarkably, there has been little evidence of geographic spread of bTB from the endemic area to date. Of the 967 culture-positive deer identified statewide among the 332,024 tested to date, 751 (78%) were from DMU 452, while 188 (19%) originated from the rest of the endemic area. Only 28 positive WTD (0.008% of all deer tested) have been identified outside those areas, 25 (89%) prior to the year 2011. This is despite statewide surveillance, which has considerably intensified, particularly in the Lower Peninsula, since 2008, due to testing for the transmissible spongiform encephalopathy chronic wasting disease, as depicted in Fig. 2B. In all but two instances (1999 in Mecosta County and 2007 in Shiawassee County) where bTB has been found in areas of the state > 70 km from the endemic area, that spread has occurred via movement of cattle, either infected in the endemic area and shipped undiscovered by testing, or infected out of state and imported into Michigan (Fig. 2A). Whole genome sequencing of *M. bovis* isolates, now carried out routinely for all infected livestock herds by USDA’s National Veterinary Services Laboratories, has considerably clarified the likely sources of infection for cattle herds, both within and outside the endemic area. Prior to WGS, if cattle tracing did not identify a likely source herd, the default assumption had been that any infected cattle herd was most likely to have been infected by local WTD, whether deer surveillance around the herd identified any infected deer or not. Now, while testing of wild deer within a 16 km radius of outlying (i.e. distant from DMU452 and the endemic area) infected cattle herds continues to occur, it primarily serves to detect spillover of *M. bovis* from those herds into the local deer population, as WGS of isolates from those infected cattle most frequently implicate genotypes already identified in other cattle herds within or outside Michigan. Thus the greatest risk of geographic spread of bTB in Michigan continues to be via livestock movement. The reader is referred to the subsequent section “Livestock: Surveillance, monitoring, oversight and policy” for additional discussion.

**Fig. 3:**
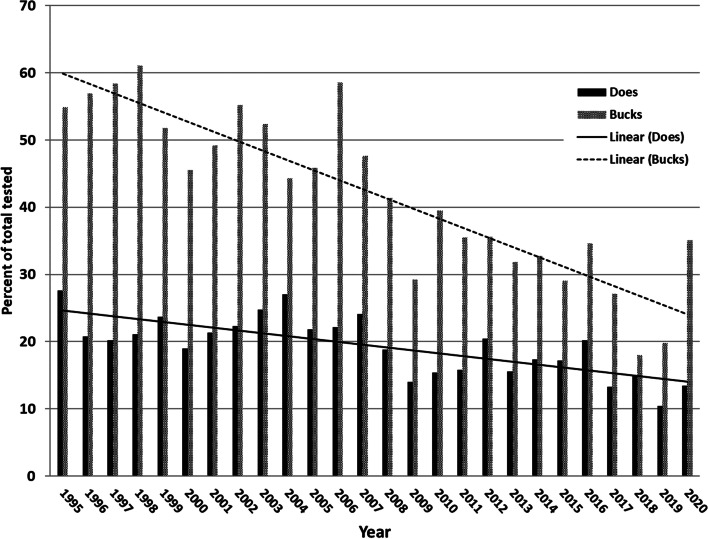
Percent of free ranging white-tailed deer tested for bovine tuberculosis that were yearlings (1 or 1.5 years old at time of death), Deer Management Unit 452, Michigan USA, 1995–2020

Previously, the prevalence of bTB in 1 year old deer provided a useful, if approximate, metric of the rate of new bTB infections in deer, using the rationale that any lesioned animals found in that age group had to have been infected within the past year [4, 5]. However, recent trends in deer hunting preferences have dramatically lowered the harvest of yearlings of both sexes, as shown in Fig. 3. Antler point restrictions (APR; prohibitions on harvest of bucks [i.e. males] with antlers below a specified size) have resulted in a shift of harvest to older animals. Such prohibitions have been voluntarily adopted by many private landowners in the endemic area in order to obtain deer with larger antlers, which are highly prized as trophies. In theory, many APR management philosophies also encourage vigorous harvest of antlerless deer (does [i.e. females] and fawns), but many landowners fail to do so, possibly because of retained taboos against doe harvest prevalent in the early twentieth century, when the deer population was still recovering from market hunting [14, 15]. Consequently, the number of yearling deer currently tested is insufficient to provide a high probability of detecting bTB, particularly in the endemic area outside the core, where bTB prevalence is considerably lower. To address this problem, attention has turned to development of force of infection (FOI) models to estimate the incidence of bTB. Rooted in the theory of catalytic epidemiological models [16], these methods employ a Bayesian hierarchical approach to model FOI as age-specific cumulative infection hazard functions over time [17, 18]. Sex, age, temporal and spatial effects are included as predictors of FOI, explicitly modeling age and time separately by following age-cohorts (e.g., yearlings) FOI over time [19]. Those models have shown that, contrary to the story told by yearling prevalence (which has been essentially flat over the past decade), bTB incidence has been steadily increasing in both sexes since at least 2012 in DMU 452, a worrying sign. In addition, the methods enable mapping of FOI over time, affording spatially explicit information valuable for both deer management and regulation of on-farm biosecurity measures.

Michigan’s highly-valued herd of free-ranging elk (*Cervus elaphus nelsoni*) remains at risk for spillover of *M. bovis* infection, and thus an ongoing concern for wildlife managers [4, 5], because of the close proximity of the population’s home range northwest of DMU 452 ([20] Fig. 1). As with WTD, surveillance testing of both hunter-harvested elk and those dying of other causes continues on an ongoing basis. To date, 4,618 wild elk have been tested for bTB since 1996, with an annual mean of 178 (S.D. = 59). Of these, 7 animals have cultured positive for *M. bovis*, 1 each in 2000,’01,’06,’12, and’17, and 2 in 2003, for an apparent period prevalence of 0.15%. That value lies within the confidence limits of the estimated true prevalence of 0.6% (0.1, 2.4) estimated previously from a limited sample [20]. Thus far, cases of bTB in elk remain rare, and there is little evidence that the species is a maintenance host in the Michigan outbreak.

#### Wildlife: baiting and feeding

Possibly no aspect of Michigan’s bTB outbreak and its management has received more attention than the provision of feedstuffs to wildlife by the public, as a putative means to increase WTD harvest (baiting) or to promote viewing and the perception of aiding population survival (recreational and supplemental feeding, resp.) [9, 12, 13, 21–23]. From the initial identification of its extent [8], bTB management recommendations for wild deer have included regulatory restrictions, or outright bans, on baiting and feeding [24]. These have been met with vocal, well-organized resistance. While such hindrance was unsurprising among deer hunters, some of the loudest opposition has come from agricultural (including livestock) producers and business owners who raise or sell crops such as carrots, sugar beets and apples for deer bait and feed [25–27]. That opposition successfully prevented any broad regulation of baiting and recreational feeding other than county by county restrictions until January 2019. At that time, the discovery of an extensive outbreak of the transmissible spongiform encephalopathy chronic wasting disease compelled implementation of a ban in the entire Lower Peninsula. The consistency of that ban across counties has aided enforcement and prosecution. It is notable, however, that over twenty years of concerted agency recommendations to manage bTB with a ban were never sufficient to bring one about, indicating the low priority of bTB management to the general public and their elected officials. A more disheartening manifestation of that low priority has been the nine bills since introduced to the Michigan Legislature to circumvent MDNR regulation and either legalize (conditionally or completely) deer and elk baiting and feeding, or reduce legal penalties for doing so to as little as US$1.00. A pair of these bills were successfully passed by both houses of the Legislature in 2019 and 2021, and required vetoes by the Governor to prevent being enacted into law [28]. Whether one graciously attributes these actions to nescience, or less charitably to a broader disregard for infectious disease epidemiology, or science in general, they nevertheless demonstrate a pervasive indifference on the part of policymakers to the history and importance of bTB control for both animal and public health. In such a policy environment, the limited progress made towards eradication so far in Michigan becomes intelligible, if not predictable.

#### Wildlife: advances due to whole genome sequencing

Beginning in 2014, a joint collaborative grant with the Universities of Glasgow, Minnesota and Wageningen and Cornell University facilitated the WGS of all the Michigan *M. bovis* isolates archived since 1994 at the National Veterinary Services Laboratories and Michigan Department of Health and Human Services Laboratory. Consisting of over one thousand sequences at the time of this writing, the sequences from wildlife isolates collected by MDNR and wildlife and livestock isolates from USDA’s Animal and Plant Health Inspection Service (APHIS), the database of Michigan sequences has enabled investigations into the genomic history of the outbreak and the role of various species in transmission, provided informative context for isolates from cattle herd breakdowns where movement records are sparse or absent, and facilitated epidemiologic insights into recent zoonotic cases of bTB.

**Fig. 4:**
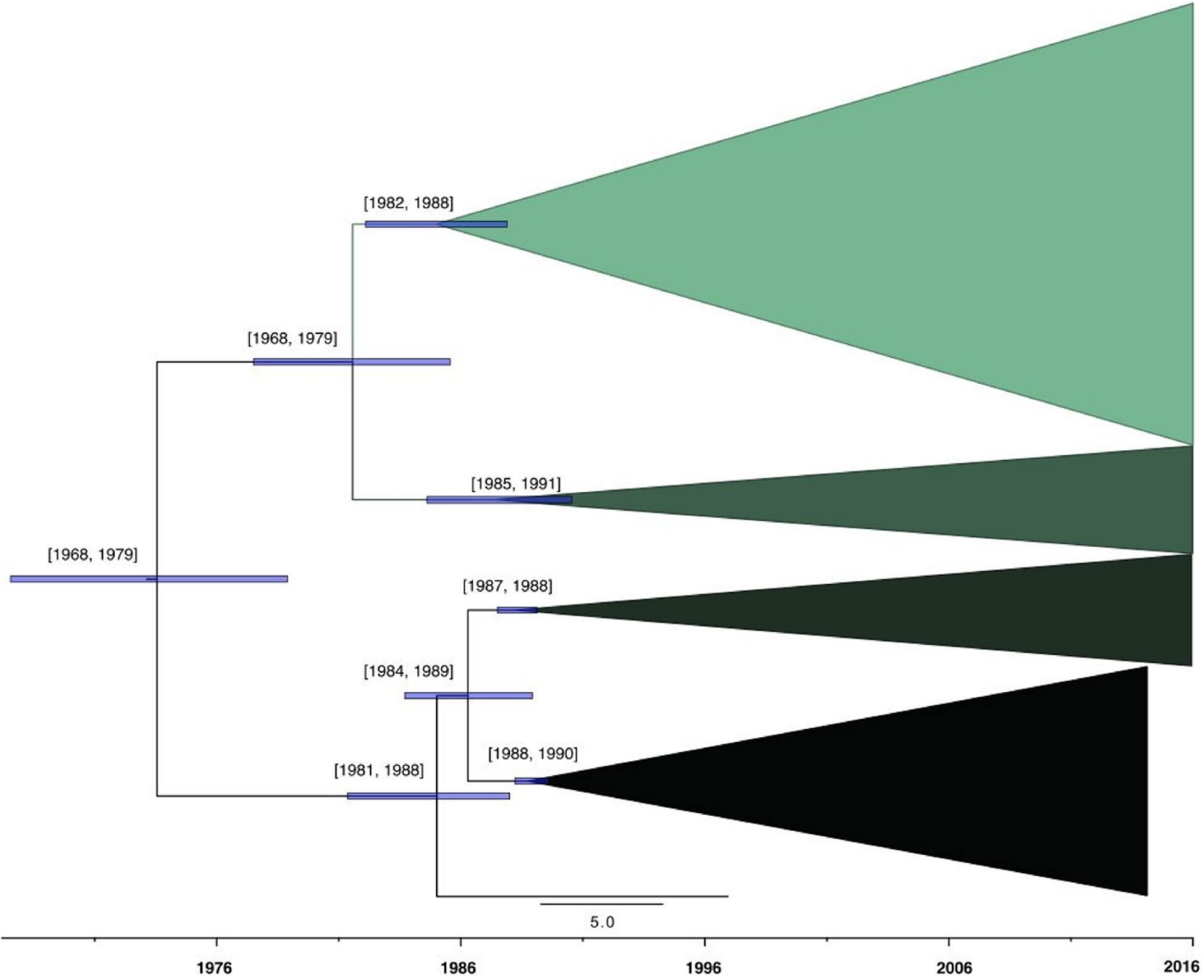
Time-calibrated maximum clade credibility tree for *Mycobacterium bovis* isolates in Michigan, USA, 1995–2020, obtained via whole genome sequencing

Owing primarily to the difficulty of observing free-ranging wildlife, understanding the emergence of infectious disease and how it has spread over space and time are frequently challenging and understudied problems. Michigan’s bTB outbreak is no exception. A recent genomic analysis [29] of WGS from 860 Michigan *M. bovis* isolates sampled 1994–2016 identified 1273 single nucleotide polymorphisms (SNPs, i.e. point mutations). Tip-date randomization tests strongly supported that these mutations are cumulative, and conserved over time. The time-measured phylogeny, estimated by Bayesian coalescent approaches, identified four divergent clades. Ancestral host-space reconstruction predicted deer as the most probable ancestral source for the current *M. bovis* lineages (albeit in the absence of WGS from cattle before 1994; see [30, 31]). None of the clades is specific to a particular sampling time, host species, nor geographic location. The clades extensively overlap spatially, with multiple host species observed in all of them. The original spillover of *M. bovis* to wild deer in Michigan (i.e. the date of the most recent common ancestor) most likely occurred about 1931 (95% HPD: [1890–1973]), the clades diverged relatively rapidly between about 1974 and 1987 as noted in Fig. 4, and have since been co-circulating independently with interspecies transmission occurring in each. This provides valuable historical context, demonstrating that bTB was already established in wild WTD for decades before control efforts were initiated, and completely differentiated by that time with all current clades extant. Given the difficulty of eradicating bTB once established in a self-sustaining wildlife reservoir, the resulting challenges of bTB management in Michigan are unsurprising. In addition, because the time period of rapid divergence of the four lineages coincided with a period of extensive supplemental feeding and baiting of wild WTD by humans, whether that feeding created genetic bottlenecks that could have shaped clade divergence warrants further investigation.

A subset of these Michigan WGS has also been used to investigate the roles of furbearers and elk in ecology of bTB. Partly because furbearing animals are maintenance hosts of *M. bovis* in the United Kingdom and New Zealand [32, 33], their hypothesized roles in Michigan have been extensively debated [34–37]. To clarify their roles, a spatial and Bayesian phylogenetic analysis was recently carried out [38] to reconstruct the bTB between-group, between-species dynamics. The study used spatiotemporal locations (1994–2016, to the 2.6 km^2^ scale) and WGSs for *M. bovis* isolated from 548 wild cervids (WTD and elk), 228 domestic animals (cattle, farmed deer and bison [*Bison bison*]), and 87 furbearers (bobcats [*Lynx rufus*], opossums [*Didelphis virginiana*], raccoons [*Procyon lotor*], coyotes [*Canis latrans*], red and gray foxes [*Vulpes vulpes* and *Urocyon cinereoargenteus*, respectively], and black bears [*Ursus americanus*]). The spatial clustering of wild and farmed animal isolates demonstrated the close proximity of wildlife to neighboring farms. Inter-group transmission events suggested by the clustering of *M. bovis* in furbearers, farmed animals, and wild cervids was verified with high statistical support. Unsurprisingly, there was high support (probability (*P*) = 0.98) for sharing (i.e. bidirectional) transmission between WTD and cattle in all four clades. At the species level, in the wild, transmission events centered on WTD, with high support of *M. bovis* sharing with coyotes (*P* = 1.0 in 3 of 4 clades). There was less support for sharing between WTD and raccoons (*P* = 0.87, 1 of 4 clades) and between coyotes and raccoons (*P* = 0.78, 1 of 4 clades), and negligible support for sharing amongst other species combinations. In a farm setting, cattle are the only species epidemiologically linked to others, with high support for *M. bovis* sharing with farmed deer (*P* = 0.78, 1 of 1 clade), and less support for sharing with opossums (*P* = 0.86, 1 of 3 clades) and raccoons (*P* = 0.78, 1 of 4 clades). Because support for transmission between WTD and raccoons and between cattle and raccoons did not occur in the same clades, the results rule out raccoons acting as a transport host for *M. bovis* between WTD and cattle. In addition, they suggest that opossums, if transmitting at all, are sharing infection only with cattle, and that farmed deer herds likely acquired bTB via sharing with cattle rather than transmission from wildlife of any species. By providing epidemiological insights from field data that would otherwise be unavailable, the study demonstrates both the theoretical and practical value of WGS studies of *M. bovis* transmission at the wildlife‐livestock interface. It also has important management implications, namely that reducing furbearer numbers is unlikely to affect cattle herd breakdowns, that farm biosecurity aimed at furbearers is likely unnecessary, other than to limit possible spillover transmission from cattle to opossums, and that white-tailed deer remain the sole maintenance host among Michigan wildlife, and farm biosecurity measures must concentrate on them.

A similar Bayesian discrete trait analysis of sequenced isolates from elk and a subsample of WTD and cattle [39] suggested that while the possibility of intermittent elk to elk transmission cannot be ruled out, elk are unlikely to be a maintenance reservoir of *M. bovis*. In addition, thus far the Michigan data do not support elk having an active role in the transmission of *M. bovis* infections to livestock populations.

The potential for zoonotic infections was one of the original drivers of the federal bTB eradication program [2], and remains important in Michigan where enzootic reservoirs currently persist. Although equivocal exposures made definitive attribution of a source impossible, human cases of *M. bovis* were reported fifteen years ago [40]. The routine sequencing of isolates from both infected cattle and wildlife has since provided a ready context for human isolates, once diagnosed. Three human cases of active bTB disease were recently tied to exposures from infected WTD [41], with isolate genotypes closely related to genotypes documented to have been circulating in deer. Three more culture-confirmed cases have also been attributed to deer [42]. One, an active cutaneous case in a taxidermist diagnosed in 2019, yielded an isolate sharing a most recent common ancestor with an isolate from an infected beef cattle herd found in Alcona County three years earlier. However, the patient’s occupation provided ample opportunity for exposure to infected deer, and the genotype of their isolate was likely circulating in deer, even though no matching genotype occurred in our database. In contrast, the most recent case was an active pulmonary infection in a young person with no history of deer hunting or cattle exposures. The WGS from that patient’s isolate was an exact match with an isolate cultured from a three year old buck harvested in Oscoda County in 2015. Follow up by public health officials found no association between the hunter and the patient. However, the patient’s family maintained a summer home approximately 32 km from the harvest location of the deer with the matching genotype. Public health investigations suggested the most likely source of the patient’s bTB infection may have been exposure to a sick fawn being rehabilitated illegally by a neighbor a number of years before. The case is perhaps most notable because it demonstrates that the risk of zoonotic infections from WTD is not limited to hunters and taxidermists. It also shows the necessity of limiting rehabilitation of deer in areas where bTB is enzootic. The third case, still under investigation by public health authorities at the time of this writing, yielded an isolate three SNPs away from a genotype recovered from three wild Alcona County deer in 2019 and 2020.

### Wildlife: Demographics and policy

For the past century, the North American model of wildlife management, which treats natural resources such as free-ranging wildlife as publicly-owned assets managed in trust for the sustained use of present and future generations, has guided both the management of wildlife populations and control of the diseases that occur in them. Control of bTB in Michigan wildlife has relied upon hunter harvest as its primary tool since the disease was first identified [8, 23]. While that reliance has kept bTB in check so far, recent demographic trends in deer hunting have the potential to profoundly affect management of the disease in Michigan in the coming years. The generation of individuals born in the years following World War II, sometimes referred to as the Baby Boomers, participate in hunting at rates considerably higher than the generations that have followed them (specifically, those born after 1980) [43, 44]. The aging, and attendant physical incapacity, of the Boomers has thus resulted in dramatic declines in WTD hunting both in Michigan and elsewhere. For example, in 1995, the first year of systematic bTB surveillance and management of WTD in Michigan, deer hunting licenses were sold to 872,000 people statewide. In 2019, 582,000 licenses were sold, a 33% decline, averaging -1.7% per year [45]. That rate of decline is expected to accelerate to -2.6% per year by 2030. Assuming this trend continues, MDNR projects that approximately 434,000 people will purchase deer hunting licenses in 2030, meaning that over half of the hunters managing deer at the outset of bTB control in Michigan will have been lost by the end of this decade. While efforts to recruit new hunters have intensified [46], minimum hunting ages have been lowered (to 12 years of age, and subsequently eliminated entirely), hunting with weapons that make harvest more likely (such as crossbows) has been expanded, and substantially more women now hunt than previously [44], none of these factors is considered likely to offset the generational effect of the aging of Baby Boom hunters. Further, these declines have been most dramatic in northern parts of Michigan, such as the bTB enzootic area.

The potential ramifications of these trends for bTB control efforts cannot be overstated. With strong evidence of density dependence on bTB transmission [23], fewer hunters will in all likelihood mean fewer deer harvested, so more deer, and consequently, greater transmission of bTB both among deer and to cattle [9, 23]. In addition, because the majority of funding for both wildlife management and disease control comes from hunting license sales, resources available for bTB management are shrinking. Recognizing these looming challenges, efforts have already begun to increase the number of deer harvested per hunter, to maintain the progress made to date. Michigan DNR has invested in a full-time bTB biologist in the enzootic area, focusing on outreach. Her efforts have concentrated on forming a Charette [47] of diverse stakeholders, and encouraging formation and growth of cooperatives (i.e. coops), groups of adjacent landowners who agree to pursue similar deer management objectives [48]. Goals for these coops are multiple. If of sufficient size, they consolidate deer management at a broader landscape scale, with a larger proportion of the population under similar management, increasing impacts. They afford fewer points for repeated contact, and a forum to educate hunters and landowners on bTB, desired control measures, and the challenges facing cattle producers, many of whom are hunters themselves. Coops also provide a target for messages on habitat management, such as shifting land use to vegetation (e.g., red pine [*Pinus resinosa*]) that supports fewer deer but is still economically valuable. In general, the focus is on building long-term trust relationships with area landowners, to increase transparency of agency decision making to the public, agency credibility, and hopefully, cooperation [49].

Yet trust does not necessarily entail increased participation in agency-directed management [50–52], and these demographic trends are likely to have effects on bTB control that extend far beyond hunting alone. Recent evidence [53] suggests that factors associated with societal modernization (such as greater urbanization, education and income) are leading to shifts in public values away from domination (beliefs that prioritize human well-being over wildlife, accept intrusive control, and justify treatment of wildlife in utilitarian terms) towards mutualism (beliefs that view wildlife in human terms, with human-like characteristics and personalities, deserving caring and compassion) [54, 55]. Increases in mutualism may facilitate important positive effects on wildlife populations (e.g., conserving biodiversity [56]). However, to the extent that they make actions resulting in death or harm to wildlife (e.g., harvest and culling) less tolerated by the public, mutualistic values may increasingly limit bTB control in deer directly, but also indirectly, as stakeholders with traditional utilitarian values (which still predominate in Michigan [57]) distrust wildlife agency actions as part of a ‘cultural backlash’ to perceived change [58, 59]. Ironically, cattle producers and deer hunters, though often at odds, may find themselves united both by their dwindling numbers [60], and their willingness to support lethal control. In the near future, WTD cattle producers and deer hunters are not able or willing to kill will likely be killed only by vehicle collisions and broader ecological factors such as disease.

#### Livestock: Surveillance, monitoring, oversight and policy

To date, there have been 81 bTB-infected cattle herds in Michigan since the first was diagnosed in Alpena County in 1998. Sixty-four of these (79%) were beef herds, with the remainder (17, 21%) dairies. The most recent positive farm was found in January 2022. Nine farms (11%) have been infected twice, and two (2.5%) three times. Seventy-eight of these farms (96%) were infected with *M. bovis* genotypes unique to Michigan (and thus could be considered native cases), while three herds were infected with genotypes related to those circulating in North American farmed elk herds for the past few decades. These three herds were infected by movement of infected cattle into Michigan from out of state. There have also been 6 feedlots infected by movement of cattle from infected Michigan source herds. Because these feedlots are considered ‘terminal facilities’ (i.e. with all animals ostensibly moving directly to slaughter from them), they are not considered in the count of infected herds by USDA. In addition, a captive bison (*Bison bison*) herd in Oscoda County was diagnosed in 2014, and 6 infected farmed deer and/or elk herds have also been identified, in 1997, 2006, 2009 (2) and 2021 (2). So far, all the captive wildlife herds sequenced were infected with Michigan genotypes.

Michigan has split state status in the USDA bTB eradication program, meaning that officially it has two bTB accreditation statuses with respect to livestock [61]. Four of the five counties where the disease is endemic in WTD (Alcona, Alpena, Montmorency and Oscoda) are zoned MA (the third level among five), while the remainder of the state is zoned AF (the highest level). Since 2010, the diagnosis of several infected cattle herds and feedlots in areas of the state that had been assumed to be free of bTB (and so subject to minimal testing and movement restrictions; relying instead on slaughter surveillance as the primary method of detection) has pointed out a vulnerability in Michigan’s bTB surveillance. Farms in the MA zone are subjected to annual whole herd tests, as well as pre-movement testing. However, such testing has not been routinely required in adjacent counties in the AF zone where bTB is either endemic, or at least periodically present, in wild deer at very low prevalence. Slaughter surveillance, while more cost effective, may not detect infected herds until several years after introduction of the disease. Consequently, covertly infected cattle originating in those areas have been moved to other areas of the state, where they have infected the destination herds, and could have seeded further infection if not discovered in timely fashion. Surveillance of WTD in those areas of origin has not (and in many cases cannot) detect the risk of cattle herd infections, because bTB prevalence in deer is so low that not enough deer can be tested to afford a high probability of detection. In addition, the demands of testing deer in other areas for chronic wasting disease have exhausted MDNR’s existing laboratory capacity. Thus, cattle testing is far more likely to be effective for detecting infected cattle herds in these low prevalence areas, be that via periodic whole herd testing or meticulous slaughter surveillance. As such, it is critical for the state to ensure that adult cattle going to slaughter are carefully inspected for granulomas, and that local inspection overseen by the state is undertaken by abattoir workers well trained to identify tuberculous lesions.

Understandably, both cattle producers and the Michigan Department of Agriculture and Rural Development (MDARD) have opposed such testing as placing onerous regulatory burdens on them, while wildlife management is not eliminating the source of *M. bovis* in free-ranging deer. That said, whole herd testing in these outlying areas has already proven efficacious, detecting an infected beef herd in Cheboygan County in 2021. At least through the end of 2023, whole herd testing of cattle (and in some cases, pre-movement testing) will be required in Presque Isle County, as well as portions of six other counties within 16 km of the MA zone border. Specific details of all requirements are available elsewhere [62]. So although those seven counties remain officially classified as AF, testing, movement and biosecurity requirements equivalent to those in effect in the MA zone have rendered them free of bTB in name only.

Maintenance of split state status requires that MDARD, MDNR and USDA negotiate and abide by (at least ostensibly) a Memorandum of Understanding (MOU) that stipulates the responsibilities each agency will carry out in order to maintain split state status. The most recent renegotiation of the MOU was signed by all three agencies in April, 2022 [62]. The provisions of the MOU are negotiated on a periodic (but variable) basis, with periodic reviews of the Michigan bTB eradication program carried out by USDA. Reports on two recent program reviews are publicly available [63]. If split state status was withdrawn by USDA, the entire state would revert to the cattle testing and movement restrictions in force in the area with the lowest accreditation status (that is, MA) resulting in considerable economic burden for cattle producers. Over the course of the bTB MOU’s history, there have been multiple instances of non-compliance, some of them marked [64, 65]. Yet, to date, USDA has not withdrawn split state status, although clearly justified in doing so under strict interpretation of the MOU. This raises the larger issue of regulatory flexibility in the USDA bTB eradication program. While commendable for sparing Michigan cattle producers the burdens of additional testing and limited markets, this flexibility may in the long run be prolonging the bTB problem. Michigan legislators and other high level decisionmakers have thus far repeatedly demonstrated their unwillingness to implement costly and unpopular, but epidemiologically necessary, disease management measures without USDA enforcing real consequences for MOU noncompliance. This has been particularly conspicuous with respect to population management of bTB in wild deer.

#### Livestock: farm biosecurity

With Michigan thus far unable (or unwilling) to eliminate bTB in WTD, considerable emphasis has been placed on cattle farm biosecurity [7]. Biosecurity requirements have been implemented by MDARD and USDA in the MA zone under two phases: Wildlife Risk Mitigation (WRM) [66], begun in 2008 [67], and Enhanced Wildlife Biosecurity (EWB) [68], initiated in January 2020. Both programs incorporated on farm bTB risk assessments by agency veterinarians, wildlife biologists and university extension personnel tailored to specific producers, and the development of farm-specific plans to reduce wildlife/livestock interactions which the producer agrees to implement, as well as periodic (typically biannual) inspections to verify that the agreed upon controls had been implemented [67]. Some controls may require added infrastructure (e.g., 2.5 m fencing of silage storage areas, covered barn storage for large hay bales, etc.) for which there is typically an agency costsharing program. Others rely on behavior modification on the part of the farmer, such as shutting barn doors and gates, or avoiding turning cattle out on pasture at times such as spring green up when deer pressure is high. The critical additional condition of EWB that producers allow targeted deer removal (i.e., culling of wild WTD) on their farms was stipulated in the 2019 MOU. Producers who refused to allow or otherwise inhibited deer removal were no longer allowed to ship live cattle except directly to slaughter.

However, many producers welcomed culling before it was required. It bears emphasis that this culling is not a population reduction strategy, but rather a tool to remove habituated deer using specific farm features that present a risk of indirect transmission of *M. bovis* to cattle at specific times of year. The USDA’s APHIS Wildlife Services branch has played a pivotal role in both programs, with primary responsibility for risk assessments, verification visits, and targeted deer removal. Since 2017, 1,890 deer have been culled from 49 farms in the designated EWB area, with another 686 deer removed from other cattle farms in the bTB area. All deer are tested for bTB at MDNR’s Wildlife Disease Laboratory, and meat from test negative deer is donated to local food charities if the farm does not keep it for personal consumption. Nine (0.3%) of these deer have cultured positive for *M. bovis* through May 2022.

A principal, if unstated, justification for implementation of farm biosecurity has been that if it proved adequate to prevent herd breakdowns, more burdensome regulation by USDA would be unnecessary. Yet previous modelling [9] has suggested that at least a 95% reduction in cattle/deer contacts would be necessary in order to reliably reduce herd breakdowns, and that biosecurity alone is unlikely to be sufficient. While endorsing the potential value of farm biosecurity, in program reviews USDA has remarked that evidence WRM or EWB are actually reducing transmission to cattle is lacking. Consequently, they have continued to require additional regulations in the MOU as well. Implementation of EWB has however provided a useful tool for producer education and outreach, and facilitated exclusion of non-verified farms, which presumably remain at higher risk, from shipping live cattle other than to slaughter.

#### Livestock: advances due to whole genome sequencing

**Fig. 5:**
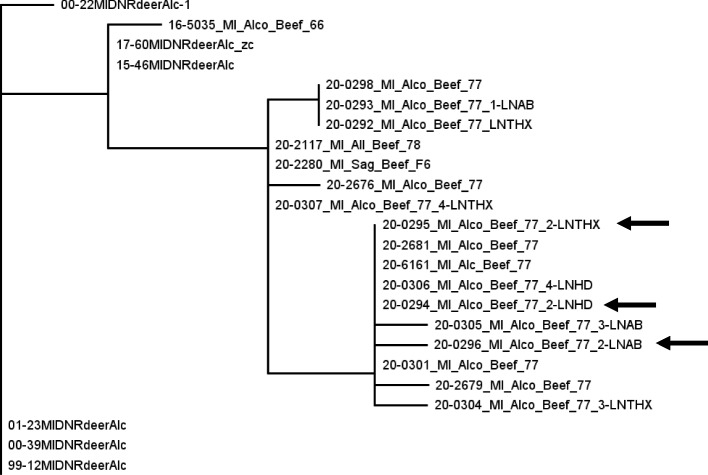
Partial phylogenetic tree of Michigan, USA, *M. bovis* isolates, illustrating genotypic variation from an Alcona County beef herd (Herd 77) first diagnosed *M. bovis* positive in January 2020. Over the course of the next eleven months, eight distinct genotypes were identified via whole genome sequencing, including two from three lymph node pools of a single animal (arrows). Genotypes identified in cattle from this herd and two infected trace out herds (Herd 78 from Allegan County and Feedlot F-6 from Saginaw County) were from three to six single nucleotide polymorphisms (SNPs, i.e., point mutations) from sharing a common ancestor with isolates collected from free-ranging white-tailed deer in Alcona County in 2015 and 2017

Although perhaps not to the extent seen with wildlife data, WGS of Michigan livestock isolates has also proven critical to facilitating epidemiological insights that were not possible previously. Prior to the availability of WGSs, when cattle herd breakdowns occurred in areas relatively distant from the bTB endemic area, if cattle movement records were insufficient to prove herd to herd transmission, or absent entirely, the default assumption was that the farm must have been infected by local deer. That initiated testing of local deer within a radius around the facility, testing which typically ended with no new infected deer being found. While also demanding more resources from MDNR field staff to contact landowners, collect deer for testing, etc., the ambiguous source of the infection considerably complicated policies for both cattle and deer management in the such areas. With WGSs, the ability to implicate herd to herd transmission due to cattle movement has clarified these situations. In addition, in the core outbreak area, WGS have provided insights on longevity of herd infections, and diagnostic failures. For example, when an Alcona County beef herd was first diagnosed as infected in January 2020, the breakdown was by default concluded to be due to recent infection from deer, because as an MA zone herd, it was subjected to whole herd testing annually. However, as the herd was subjected to repeated tests and additional infected animals were found, considerable genotypic diversity was documented, as noted in Fig. 5. In total, eight distinct but closely related genotypes were identified. Initially, the breakdown was attributed to multiple separate infection events from WTD. However, as data accumulated, it became evident that the diversity was more likely due to cattle to cattle transmission within the herd that had likely been going on for years (given the mean Michigan *M. bovis* evolutionary rate of 0.37 substitutions per year [39], about one mutation every 2.7 years), all while the herd was being tested repeatedly without being diagnosed as *M. bovis* positive.

### The devil you don’t: Finishing the job of bTB eradication in the U.S.

#### Wildlife: Other states

Outside of Michigan, the only remaining state with endemic bTB in wildlife is Hawaii, on the island of Molokai, presumed to be maintained in feral swine [69, 70]. The state was declared AF in 1993 following a complete depopulation of cattle on Molokai in 1985. However, infected cattle were subsequently found in 1997 and 2021, followed by infected domestic swine (at high prevalence) later in 2021. All those herds were depopulated. Additional livestock herd breakdowns occurred in the first four months of 2022, culminating in island-wide movement restrictions for all ungulates other than horses [71]. The source of the current outbreak remains under investigation. There is speculation that wildlife is involved, and that ongoing drought has increased contact with livestock.

The occurrence and elimination of a bTB outbreak in cattle and free-ranging WTD in Minnesota has been described elsewhere [72, 73]. No additional infected cattle or wildlife have been diagnosed there in a decade. The marked contrast in outcomes compared to Michigan’s ongoing outbreak is likely attributable to the rapid diagnosis of bTB in Minnesota, evidenced by the limited genotypic diversity of *M. bovis* isolates there [73], although sociopolitical differences played an important role as well [74, 75].

#### Livestock: Nationwide surveillance and epidemiology

Excluding Michigan and Hawaii, from 2014 through 2021, the annual number of infected cattle herds in the US has remained low among both dairies (μ = 1.5, σ = 1.3, range: 0–4) and beef herds (μ = 1.5, σ = 1.2, range: 0–4). Over the last two decades, the distribution of infected livestock herds by type has been 57% beef, 34% dairy and 9% farmed cervid. While the absolute number of infected herds per year is small, it is notable that, compared to Michigan, the average size of these infected herds is orders of magnitude higher.

**Fig. 6:**
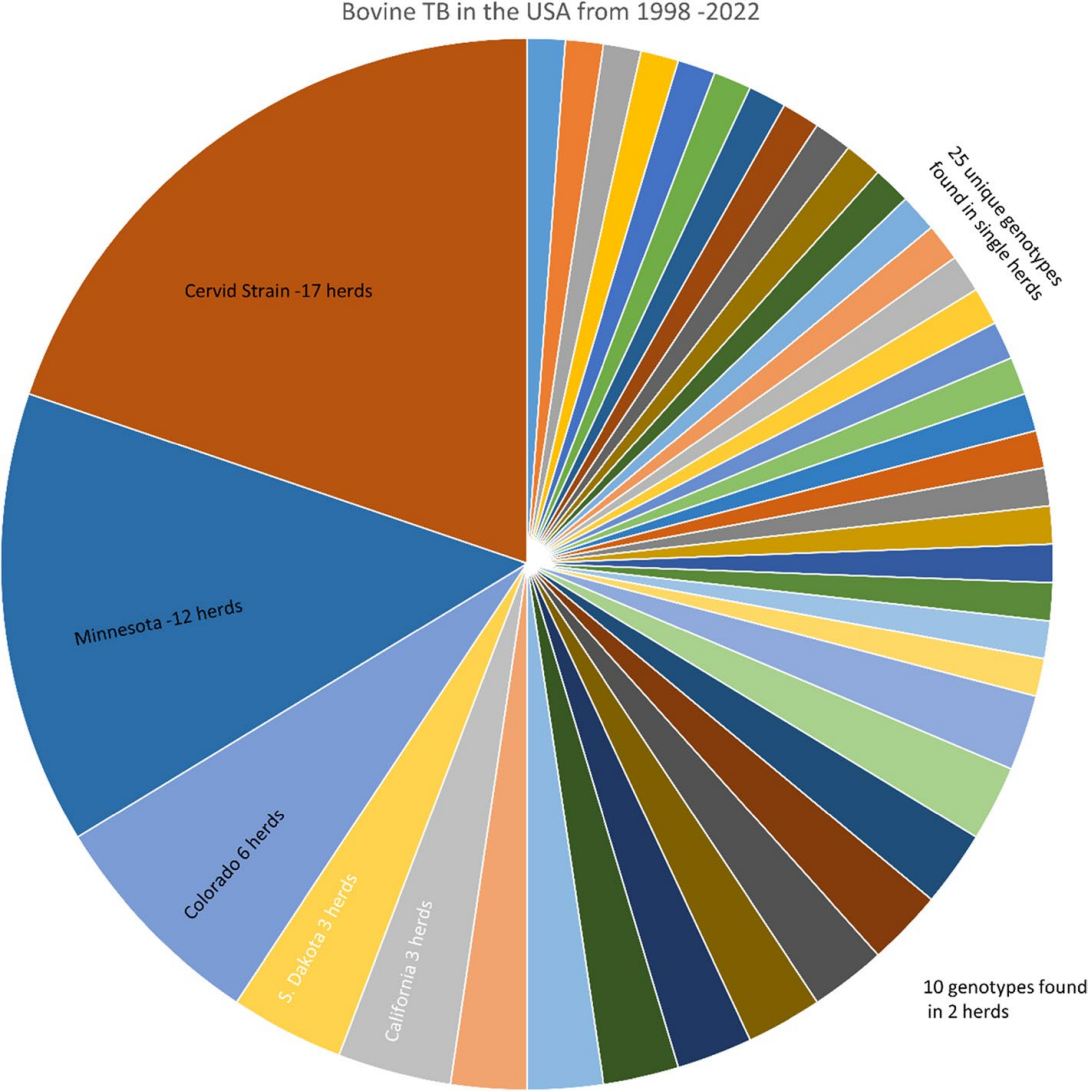
Number of cattle herds infected with a *M. bovis* genotype in the United States, excluding Michigan, 1998–2022, determined via whole genome sequencing. Genotypes from 95 herds were available for this analysis

The epidemiology of bTB in the US outside of Michigan and Hawaii can perhaps be best characterized as consisting of a low level of new introductions of genotypes of Mexican or unknown origin arising in both beef and dairy cattle, with minimal subsequent herd to herd transmission. In contrast to much of the US’ bTB eradication history, it is now extremely rare to discover an infected herd that traces to an infected source herd, or any affected herds at all. Whole genome sequencing of isolates from cases detected at slaughter from 2014–2021 has, with only a single exception, revealed previously unrecorded genotypes predominantly of Mexican origin, as shown in Fig. 6. Typically, about 45% of the total US cases annually are detected by passive slaughter surveillance. The ensuing epidemiological investigations of those detect another 36% of total cases, after which that specific genotype is never seen again. Thus, the US seems to somehow be regularly allowing entry of new bTB strains of chiefly Mexican or unknown origin, rather than covertly maintaining an ongoing reservoir of known domestic WGSs that continue to infect US cattle herds at a very low level. However, the proximate source of entry of these genotypes is unclear. Although there has been a high demand for imported Mexican-origin cattle to be fed to slaughter weight in the US (hereafter, feeders; steers: μ = 849,912 per year, σ = 162,111; spayed heifers: μ = 209,356 per year, σ = 101,352) over that period, the number of infected Mexican-origin feeder cattle detected at slaughter remains very low (μ = 3.3 per year, σ = 1.3). By regulation, all of these imported feeders must be skin test negative in order to gain entry to the US. In contrast, the number of mature, US origin, cull cattle detected at slaughter as infected with predominantly Mexican genotypes previously unrecorded in the US has actually increased over the same period (μ = 4.1 per year, σ = 1.9). Typically, these culls are from closed herds with good biosecurity.

So what might the source(s) of these new genotypes be? Several hypotheses exist. Given the low sensitivity of slaughter surveillance (assumed by USDA to be approximately 30%), it is plausible that some infected cattle are being missed at slaughter. Yet, the genotypes are overwhelmingly novel to the US, and as bTB control in Mexico has progressed markedly over the past two decades, Mexican origin bTB cases detected at slaughter have declined exponentially since 2001. Whether the speculated number of missed infections would provide sufficient foundation for these ongoing novel genotypes is debatable. Moreover, covert infections in feeder cattle do not appear to explain the pool of infections detected in mature, US-origin culled cattle. There is a possibility that those cases could be reverse-zoonotic in origin, as WGS has documented transmission of bTB from livestock workers to cattle in the US previously [76]. How common such infections are is unclear however, as is whether they occur with sufficient frequency to account for the ongoing infections detected in cull cattle at slaughter. Investigation of reverse-zoonotic infections is also complicated by the transience and mobility of those workers, and by privacy protections for patient health information that are now standard operating procedures for US public health agencies. Another source of infections is imported ‘event’ cattle for rodeos, sometimes referred to as ropers, which come into the US regularly in small numbers as test negative imports. These cattle work events and after retirement effectively disappear as they are moved and shared in the private sector before being sent to slaughter. It is plausible that these event animals occasionally manifest infection after importation, and because of their movement through private channels, their potential to expose US cattle is greater than for imported feeder cattle. Finally, the possibility of latent infections undetectable with commonly used bTB diagnostics cannot be dismissed. It is clear that latent bTB infections occur in other species. If they also occur in cattle, this could suggest that any trading with endemically infected countries necessarily carries some risk.

Amidst this ambiguity, USDA has provided funding for pilot projects that may help identify potential solutions. Firstly, radiofrequency individual animal identification (RFID) infrastructure is being installed in Texas cattle markets that process most of the Mexican-origin feeder cattle prior to slaughter. The RFID approach has been one of the mutually praised success stories of the bTB eradication program in Michigan, and the hope is that it will facilitate traceability of imported cattle as well. That capability could prove valuable to both the US (to identify existing weaknesses in the system, situations where beef and roping cattle are interacting with dairy cattle, and help design a resilient agricultural system), and to Mexico (to identify disposition of their cattle). It could also provide a marketing tool aimed at consumers, showing that the beef they eat can be accounted for from ‘farm to fork’. Secondly, additional meat inspection personnel are being placed at US abattoirs that slaughter many Mexican-origin cattle. Line speed at modern facilities is so rapid (200–400 head slaughtered per hour [77]) that it is effectively impossible for meat inspectors to closely examine all carcasses without slowing processing unacceptably. While unlikely to eliminate missed lesioned cattle, more inspectors can only improve current detection rates. Thirdly, a pilot cattle vaccination study is underway in Mexico. The most widely studied vaccine for bovine tuberculosis is the human tuberculosis vaccine (Bacille Calmette-Guérin, BCG) created from an attenuated strain of *M. bovis*. Estimates of vaccine efficacy vary widely depending on the study; however in all studies, *M. bovis* BCG consistently decreases disease severity even though it does not necessarily prevent infection. Most of the published BCG efficacy studies have focused on measures of individual animal protection but a recent meta-analysis [78] suggested that although individual animal immunity may be low, over time, the herd immunity effects of BCG vaccination can be significant. To examine the efficacy of BCG vaccination on a herd level, USDA APHIS has initiated a prospective, randomized, double-blinded trial of BCG vaccine efficacy in commercial dairies in the Mexican state of Baja California. Bovine tuberculosis is endemic in Baja California, which is classified as a Level 5, non-accredited region, meaning that cattle from Baja California cannot be exported to the US. The 5-year study will be conducted using four privately-owned modern dairies, dedicated to commercial milk production. Measures of vaccine efficacy will include estimates of disease severity obtained at postmortem examinations of culled cows, time to positivity using various diagnostic assays, and the incidence rate of infection between vaccinated and placebo animals. Investigations into *M. bovis* shedding in nasal secretions and milk, use of alternative diagnostic strategies to differentiate infected from vaccinated animals and the effects of vaccination on production and comorbidities will also be examined. While unlikely to directly improve detection of covertly infected cattle in the US, measures that continue to decrease incidence and persistence of bTB in Mexico will benefit both countries.

#### Livestock: policy challenges

As the cattle industry in the US has changed, a key tool of the bTB eradication program, herd depopulation, has become obsolete in many cases. USDA APHIS Veterinary Services (VS) budget for funding producer indemnity for condemned herds has not kept pace with growth in herd sizes, some of which are now in the 75,000 to 100,000 head range, as the industry pursues economies of scale. For example, in 2021, VS’ entire budget for indemnity was US$1 million, equivalent to about €955,000. When assessing disposition for a recent infected Wisconsin dairy of 5,000 head, if the maximum indemnity reimbursement of US$3,000 (€2,836) were paid for every animal in the herd, the total cost to depopulate this one herd (US$15 million, or about €14.3 million) would have exceeded the entire annual VS indemnity budget by 15 times, despite it not even being ‘large’ by US standards. Avenues do exist for VS to solicit the US Congress for supplemental appropriations to bolster the indemnity budget. However, the current polarized and contentious political climate in the US portends a low probability of success for such requests. In addition, the legislature is unlikely to fund supplemental appropriations repeatedly, so the circumstances where such funds are sought need to be considered carefully by VS decisionmakers. Finally, the increased size of US dairy cattle herds also means that multiple loans may be required to assemble a large herd. If different banks have liens against an infected herd, decisions to depopulate are even further complicated, as ‘ownership’ of the herd is not straightforward.

More generally, although regulatory flexibility is often sought by cattle producers for infected herds both in Michigan and nationwide, there are clearly tradeoffs between regulatory flexibility and regulatory efficacy. Inevitably, some consistency in regulation is necessary. If the eradication program is not actually making consistent progress towards eradication, justification for its very existence comes into question.

#### Livestock: advances due to whole genome sequencing

The use of whole genome sequencing has revolutionized epidemiologic investigations at both the national and herd level. In addition to identifying *M. bovis* genotypes not previously observed in the US and ruling out herd-to-herd transmission, WGS has also facilitated evaluation of on-farm transmission at a level of detail impossible just a few years ago. Two recent herd investigations showcase this.

The first, an 8,000 cow herd was detected as infected in 2018 during a herd test which was required because cattle had been purchased from another herd that was subsequently found infected with *M. bovis*. From 75 cows found infected, WGS identified three different *M. bovis* genotypes – 6A, 13A and 17B1 – none of which matched the genotype found in the source herd. One cow was found infected with 6A, one with 13A and the rest with 17B1. Whole genome sequences of the cows with 17B1 showed more than 50 cows having the exact same genotype, suggesting a common source of exposure. Most of the cows infected with the 17B1 strain calved within a 2-month period. On-farm investigation suggests the exposure occurred in the calving area. The remaining 17B1 isolates showed no evidence of cow-to-cow transmission since there were no additional accumulated SNPs shared among isolates from different cows. From this analysis, the WGS data suggested at least three different exposures for each *M. bovis* strain and a common source exposure, possibly human, for the cows with the 17B1 isolates.

The second investigation was on a multi-site facility in New Mexico with more than 10,000 dairy cows and heifers. The dairy was undergoing implementation of a test and remove bTB eradication plan and had undergone multiple tests, with the number of newly infected cows decreasing with each subsequent test. On the sixth whole herd test, nine first lactation heifers were detected as infected. An on-farm investigation into the housing of these nine heifers and previously detected infected cows identified the close-up dry pen as the place of exposure. The WGS of the isolates from the infected heifers showed a common source exposure to one of the infected cows being housed in the same close-up pen.

These two herd investigations, using WGS, highlight the time around calving as a risky period for *M. bovis*-infected cows to shed and expose their pen mates. Because dairy cattle are immunosuppressed around the time of calving, it makes sense that infected cows may lose control of the infection and shed during this time and that non-infected cows may be more susceptible to infection.

## Conclusions

Since bTB was first diagnosed in Michigan in 1975, a great deal has been learned about the pathogenesis and ecology of *M. bovis* in a North American deer-cattle system. Response to the outbreak has prompted innovations such as radiofrequency identification of individual cattle and much improved farm biosecurity, and facilitated collection of epidemiological data that are unique in the world. Moreover, the geographic scope of the outbreak has remained limited to a relatively small area, and prevalence of the disease in multiple species has been constrained. However, Michigan’s often-stated goal of “eradicating bovine tuberculosis in Michigan deer” [79] remains far from realization. Changing demographics, as well as apathy and antagonism on the part of the public and their elected officials, do not inspire confidence that this stated goal will ever be reached. Thus, Michigan finds itself on a path of perpetual management, unwilling to devote resources and political will necessary to get rid of bTB, or in some cases, to even recognize it as a serious problem. Ironically, the current approach of accreditation zoning and split state status may be an unwitting contributor. While zoning allows large areas of a state without evidence of *M. bovis* infection to carry on normally and avoid the costs of disease control, as the MA zone shrinks to the smallest defensible area, the number of people subject to the pains of bTB shrinks, arguably to the point that their political clout is no longer sufficient to mount any meaningful progress towards eradication. It also magnifies the potential influence of non-cooperators [80]. While research towards solutions continues [7], ultimately, if Michigan is content to tolerate bTB as an inconvenient but acceptable characteristic of its publicly-owned natural resources, then no end is in sight.

In the rest of the US, it has been argued by both cattle producers and agency staff alike that the current nationwide bTB regulatory structure is no longer likely to result in eradication. But with current weaknesses in the regulatory system as yet poorly defined, how the US should restructure the eradication program to best address them and move forward is also unclear.

## Data Availability

Data sharing is not applicable to this article as no datasets were generated or analyzed during the current study.

## Abbreviations

AF: Accredited Free
APHIS: Animal and Plant Health Inspection Service
APR: Antler point restrictions
bTB: Bovine tuberculosis
BCG: Bacille Calmette-Guérin
DMU: Deer Management Unit
EWB: Enhanced Wildlife Biosecurity
FOI: Force of infection
MA: Modified accredited
MDARD: Michigan Department of Agriculture and Rural Development
MDNR: Michigan Department of Natural Resources
MOU: Memorandum of Understanding
RFID: Radiofrequency individual animal identification
SNP: Single nucleotide polymorphism
US: United States of America
USDA: United States Department of Agriculture
VS: Veterinary Services
WGS: Whole genome sequencing
WTD: White-tailed deer
WRM: Wildlife Risk Mitigation
